# Iron deficiency aggravates hepatic inflammation in suckling piglets via endoplasmic reticulum stress-driven NF-κB pathway activation

**DOI:** 10.1186/s40104-026-01356-4

**Published:** 2026-02-13

**Authors:** Jun Qi, Yaxu Liang, Dongming Yu, Weite Li, Fei Long, Meng Yuan, Zhangbo Lou, Chunxue Liu, Gaiqin Wang, Bencheng Wu, Xiang Zhong

**Affiliations:** 1 College of Animal Science and Technology, Nanjing Agricultural University, Nanjing, Jiangsu 210095 China; 2 Institute of Animal Husbandry and Veterinary Science, Shanghai Academy of Agricultural Sciences, Shanghai, 201106 China; 3 Institute of Animal Nutrition and Health Industry, Anyou Biotechnology, Nanjing Agricultural University, Nanjing, Jiangsu 210095 China; 4 The College of Animal Science and Technology and College of Veterinary Medicine, Huazhong Agricultural University, Wuhan, Hubei 430070 China; 5 National Reference Laboratory of Veterinary Drug Residues (HZAU) and MAO Key Laboratory for Detection of Veterinary Drug Residues, Huazhong Agricultural University, Wuhan, Hubei 430070 China; 6 Natural Plant and Animal Health Innovation Institute, NJAU-Cohoo Biotechnology, Nanjing Agricultural University, Nanjing, Jiangsu 210095 China; 7 State Key Laboratory of Meat Quality Control and Cultured Meat Development, Nanjing Agricultural University, Nanjing, Jiangsu 210095 China

**Keywords:** Endoplasmic reticulum stress, Hepatic inflammation, Iron deficiency, Piglet, TLR4/NF-κB pathway, Unfolded protein response

## Abstract

**Background:**

Iron deficiency (ID) poses a significant health burden to both human infants and suckling piglets. In piglets, ID leads to substantial economic losses for the industry by compromising growth performance, health, and survival. However, current research has predominantly concentrated on hematological abnormalities, whereas the mechanisms underlying ID-associated hepatic inflammatory injury remain inadequately elucidated. Our study employed the iron-deficient suckling piglet model to address this knowledge gap and to establish a molecular theoretical foundation.

**Results:**

To investigate the underlying mechanisms, this study conducted in vivo and in vitro models. In piglets, ID triggered hepatic oxidative stress by inducing a redox imbalance and suppressing the core Nrf2/HO-1 antioxidant signaling pathway. Histopathological examination revealed structural abnormalities in ID piglet livers, including disorganized hepatic cords, cytoplasmic vacuolation, hydropic degeneration, and mononuclear inflammatory cell infiltration. Transmission electron microscopy further showed shrunk nuclear envelopes, reduced numbers of rough endoplasmic reticulum (RER), and dilated RER cisternae in hepatocytes of ID piglets. Mechanistically, ID activated endoplasmic reticulum stress (ERS) and the PERK/IRE1α branches of the unfolded protein response (UPR). RNA-seq transcriptomic analysis demonstrated significant dysregulation of immune-related pathways, accompanied by elevated pro-inflammatory cytokines (e.g., *IL1B*, *TNF*) and decreased anti-inflammatory cytokines (e.g., *IL4*, *IL10*). Central to this inflammatory response was the activation of the TLR4/NF-κB pathway, evidenced by upregulation of MyD88 and increased phosphorylation of IκBα and NF-κB p65. In vitro, deferoxamine (DFO)-induced ID in AML12 hepatocytes consistently recapitulated the key features of this phenotype, including the activation of ERS/ UPR and the TLR4/NF-κB signaling pathway. Pharmacological inhibition of ERS by 4-phenylbutyric acid (4-PBA) attenuated DFO-induced NF-κB activation and ameliorated the imbalance between pro- and anti-inflammatory cytokines.

**Conclusions:**

ID exacerbated hepatic inflammation through ERS-mediated activation of the NF-κB pathway, providing novel mechanistic insights into liver injury associated with ID.

**Graphical Abstract:**

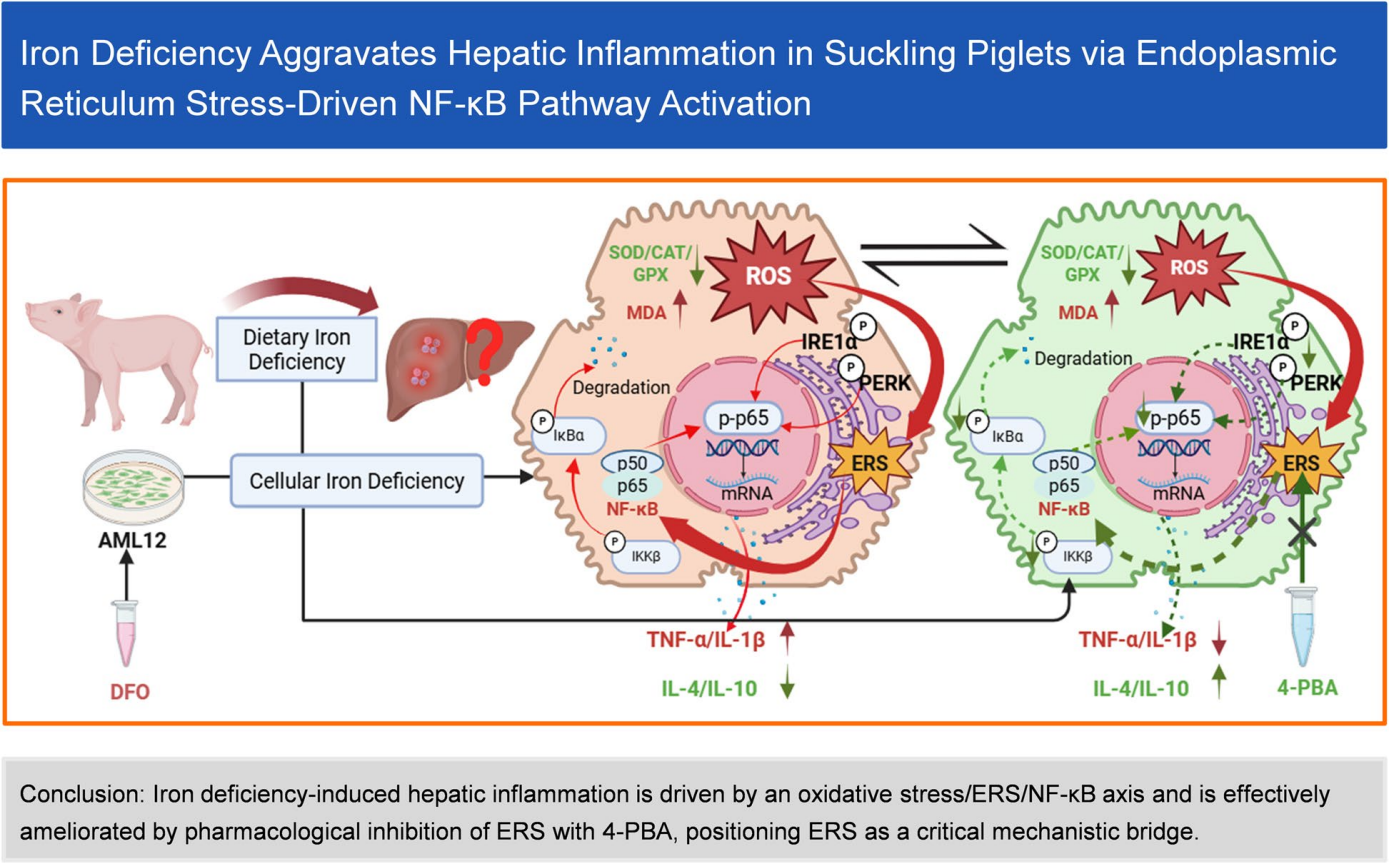

**Supplementary Information:**

The online version contains supplementary material available at 10.1186/s40104-026-01356-4.

## Introduction

Iron is an essential trace element for the growth and development of humans and animals [1]. Iron plays a crucial role in diverse physiological processes, such as oxygen transport and storage, cellular signal transduction, energy metabolism homeostasis, and immune defense mechanisms [2]. Despite its importance, iron deficiency (ID) remains the most prevalent nutritional disorder worldwide, posing a significant health burden to infants and young children. In the pig production system, neonatal piglets are generally congenitally deficient in iron reserves, with a total iron reserve of 30–50 mg at birth. Suckling piglets have a significant growth advantage (a daily weight gain of 200–300 g), which requires 7–16 mg of iron daily [3]. However, the milk iron content of suckling piglets is insufficient. Therefore, the iron obtained by neonatal piglets through breast milk can only meet 6.25%–14.29% of their physiological needs [4]. The intensive breeding mode results in a significant increase in litter size caused by modern genetic breeding techniques, the iron metabolism system of neonatal piglets is not yet mature, and the technical flaws (such as insufficient dosage or improper application timing in the conventional iron supplementation regimens). These factors collectively exacerbate the incidence of iron deficiency anemia (IDA) in neonatal piglets. This pathological state also impedes the development of immune organs in suckling piglets, clinically causing growth retardation, diarrhea, oxidative stress and immune deficiency [5]. Therefore, ID remains an important nutritional and metabolic issue in pig production.

The liver is the principal organ for iron storage and metabolism, responsible for hepcidin synthesis and ferritin storage [6]. It also acts as a key immunological hub, integrating innate and adaptive immunity to clear pathogens, modulate inflammation, and maintain oxidative balance [7]. However, most of the existing studies have focused on the effects of ID on immune cells in the intestine, spleen, and peripheral blood [8–10], while its effect on liver inflammation in suckling piglets remains poorly defined at the molecular level.

The endoplasmic reticulum (ER) is a pivotal organelle responsible for maintaining cellular homeostasis by orchestrating protein folding, assembly, trafficking, and intracellular calcium balance [11]. Under pathological or stress conditions, such as energy deprivation, oxidative stress, or calcium overload, protein folding fidelity is compromised, leading to the accumulation of misfolded or unfolded proteins within the ER lumen [12–14]. This overload triggers endoplasmic reticulum stress (ERS), a conserved adaptive mechanism. The liver, owing to its high metabolic activity, is particularly susceptible to ERS. Emerging evidence has established ERS as a critical nexus linking nutritional deficiencies to tissue pathology, with its role in diverse pathophysiological processes gaining prominence [11, 15]. Mechanistically, ERS activates the UPR signaling pathways, including protein kinase R-like endoplasmic reticulum kinase (PERK), inositol-requiring enzyme 1α (IRE1α), and activating transcription factor 6 (ATF6), which initially promote adaptive gene expression and inflammatory modulation to restore proteostasis [16]. However, chronic or unresolved ERS drives cellular dysfunction and apoptosis [16]. In enterocyte-specific ferroportin knockout mice, chronic ID markedly elevates ERS markers, including glucose-regulated protein 78 (GRP78) and C/EBP-homologous protein (CHOP), and impairs autophagic flux [17]. Similar ERS perturbations under iron deprivation have been reported in cultured cardiomyocytes and neuroblastoma cells [18, 19]. Despite these advances, it remains unclear whether ID directly induces hepatic ERS in suckling piglets and the underlying molecular mechanisms have yet to be elucidated.

Owing to the high prevalence and adverse outcomes of ID in both human infants and suckling piglets, and given that the mechanisms linking ID to hepatic ERS and inflammation remain poorly defined, this study was designed to investigate these processes using the suckling piglet model. This model is highly relevant due to its strong physiological parallels with human infants, including liver function, gastrointestinal development, and metabolic maturation. Our work aims to establish a molecular-level theoretical basis for refining iron supplementation and designing targeted therapies to prevent ID-associated liver injury.

## Materials and methods

### Animal and feeding management

The experimental procedures for piglets have been approved by the Animal Ethics Committee of Nanjing Agricultural University (License No.: SYXK-2017-0027). Piglets were obtained from five Landrace × Yorkshire crossbred sows (third parity, litter sizes of 11–13), with contemporary farrowing dates (within 3 d). After two days of breastfeeding, four male piglets per litter (birth weight: ~ 1.72 kg ± 0.5 SD) were selected and randomly divided into two groups (*n* = 10 piglets/group): The normal control group (NC) received intramuscular iron dextran (40 mg Fe/kg body weight) on postnatal d 3 and d 14; while the iron-deficient group (ID) was administered phosphate-buffered saline (PBS). Both groups were fed an iron-deficient milk formula (the composition in Table S1) [20]. During the feeding process of piglets, radiant heaters were utilized to provide supplemental heat from 07:00 to 19:00, ensuring 12 h of daily light exposure. The temperature was maintained as follows: 30–32 °C for the first three days, 28–30 °C from d 4 to 7, 25–27 °C from d 8 to 15, and 22–24 °C from d 16 to 21. The relative humidity was kept between 65% and 75%. Detailed feeding schedules are outlined in Table S2. Six piglets per group were randomly selected and euthanized on d 21.

### Cell culture

The AML12 (alpha mouse liver 12) mouse hepatocyte cell line was obtained from Nanjing Yifeixue Biotechnology Co., Ltd. (Cat No. CL-0602, China). The cells were cultured in DMEM/F12 medium (Cat No. 11320033, Gibco, Carlsbad, CA, USA) supplemented with 10% fetal bovine serum (FBS), 1% penicillin/streptomycin solution, 1% insulin-transferrin-selenium (ITS) supplement, and 0.1% dexamethasone. At approximately 70% confluence, cells in the deferoxamine (DFO) group were treated with 300 μmol/L of deferoxamine.

### Detection of iron content in liver tissue

Hepatic iron content was determined using a commercial Iron Assay Kit (Cat No. A039-2-1, Jiancheng Bioengineering Institute, Nanjing, China) according to the manufacturer's instructions. Briefly, approximately 100 mg of liver tissue was homogenized in a ninefold volume of normal saline. The homogenate was then mixed with an acid solution and a reducing agent provided in the kit. This treatment liberates iron from binding proteins and reduces all ferric iron (Fe^3+^) to the ferrous form (Fe^2^^+^). The reduced iron subsequently chelates with 2,2'-bipyridine to form a stable pink-colored complex. The absorbance of the complex was measured at a wavelength of 535 nm using a microplate reader. A standard curve with known iron concentrations was run concurrently to quantify the iron content in each sample.

### Enzyme-linked immunosorbent assay (ELISA)

Hepatic reactive oxygen species (ROS) levels were indirectly assessed by quantifying 8-hydroxy-2'-deoxyguanosine (8-OHdG), a stable and specific DNA adduct formed by hydroxyl radical-mediated oxidative damage [21], using a porcine-specific ELISA kit (AiDiKang Biotechnology Co., Ltd., Wuhan, China; Cat No. AD51838). Briefly, liver specimens were weighed and homogenized in ice-cold PBS (pH 7.4) at a 1:9 (w/v) ratio. The homogenates were snap-frozen in liquid nitrogen and stored at −80 °C. For analysis, samples were thawed at 2–8 °C and then re-homogenized in PBS, followed by centrifugation at 2,500 × *g* for 20 min at 4 °C. The resulting supernatants were aliquoted for immediate use or storage.

For cytokine measurement, frozen liver tissues were thawed at 2–8 °C and homogenized in PBS (1:9, w/v; supplemented with protease inhibitors) using a high-speed homogenizer (three cycles of 30 s at 25,000 × *g*, with intervals in an ice bath). The homogenates were then centrifuged at 5,000 × *g* for 10 min at 4 °C. The supernatants were collected, aliquoted, and stored at −80 °C until analysis. Concentrations of interleukin-1 beta (IL-1β) and tumor necrosis factor-alpha (TNF-α) were determined using commercial porcine ELISA kits (Jiancheng Bioengineering Institute, Nanjing, China; Cat No. MBE10289 and MBE10037, respectively), strictly following the manufacturers' instructions.

### Hepatic oxidative stress

For each sample, 100 mg of piglet liver tissue was accurately weighed and then mechanically homogenized with normal saline at a 1:9 (w/v) ratio in an ice-water bath to prepare a 10% (w/v) homogenate. The homogenate was centrifuged at the speed specified by the kit instructions, and the supernatant was collected for analysis. The activities of total superoxide dismutase (T-SOD), catalase (CAT), and glutathione peroxidase (GSH-PX), as well as the concentrations of reduced glutathione (GSH) and malondialdehyde (MDA), were determined using kits provided by Nanjing Jiancheng Bioengineering Institute, China.

### Cell viability assay

The viability of AML12 cells was assessed using the Cell Counting Kit-8 (CCK-8) assay (BS350B, Biosharp, Nanjing, China). Cells in the logarithmic growth phase were trypsinized and resuspended in complete medium to achieve a density of 1 × 10^4^ cells/mL. The cell suspension was seeded into 96-well plates at 100 μL/well and incubated for 24 h to allow cell attachment. Following adhesion, the original medium was replaced with 100 μL of a working solution containing 4-phenylbutyric acid (4-PBA) at different concentrations (0, 0.1, 0.5, 1, 2.5, and 10 mmol/L). Six replicates were set up for each concentration, along with blank controls containing only medium. Following 24 h of incubation, 10 μL of CCK-8 reagent was added to each well according to the manufacturer's protocol. The plates were then gently mixed and incubated at 37 °C for 30 min to 1 h in the dark. Absorbance was measured at 450 nm using a microplate reader, with 630 nm as the reference wavelength to correct for measurement errors. Each experiment was conducted in triplicate.

### Total RNA extraction and quantitative real-time PCR (qRT-PCR)

Total RNA was extracted from liver tissues or cells using TRIzol™ reagent (Invitrogen, Carlsbad, CA, USA). RNA concentration and purity (A_260_/A_280_ ratio ≥ 1.8) were quantified using a NanoDrop™ 2000 spectrophotometer (Thermo Fisher Scientific, Waltham, MA, USA). After normalizing RNA samples to 500 ng/μL with RNase-free water, 2 μL RNA was reverse-transcribed into cDNA using a PrimeScript™ RT reagent kit (RR047A, Takara Bio, Dalian, China) following manufacturer's instructions. qRT-PCR reactions were performed in 10 μL volumes using SYBR^®^ Green Master Mix (11718, Accurate Biotechnology, Changsha, China) with the following thermal cycling parameters: initial denaturation at 95 °C for 5 min; 40 cycles of 95 °C for 10 s and 60 °C for 30 s; followed by dissociation curve analysis (95 °C for 15 s, 60 °C for 1 min, and 95 °C for 15 s). Relative mRNA expression levels were calculated using the 2^−ΔΔCt^ method, using *GAPDH* as an internal housekeeping gene. The primers were designed using NCBI/Primer-BLAST. The primer sequences and gene accession numbers were listed in Tables S3 and S4.

### Western blot

Total proteins were extracted using RIPA lysis buffer supplemented with PMSF and phosphatase inhibitors (100:1:1, v/v). Protein concentrations were quantified by BCA assay (Beyotime Biotechnology, P0010) and adjusted to 1 μg/μL. Samples were mixed with 5 × SDS loading buffer at a 4:1 ratio and denatured at 100 °C for 5 min. Proteins were separated by SDS-PAGE (150 V, 45 min) and transferred to PVDF membranes (300 V, 45 min). The membranes were blocked with 5% skim milk/TBST for 2 h at room temperature. Sequential incubations were performed with primary antibodies (overnight at 4 °C) and HRP-conjugated secondary antibodies (1 h at room temperature), followed by three 10 min TBST washes after each antibody incubation. Protein bands were visualized using ECL chemiluminescent substrate under an ultrasensitive chemiluminescence gel imaging system. Quantitative analysis was conducted with ImageJ software. Detailed antibody information is listed in Table S5.

### Hematoxylin and eosin staining

Fresh liver specimens were fixed in 4% paraformaldehyde (PFA) for 24 h at 4 °C, followed by graded ethanol dehydration (70%, 85%, 95%, and absolute ethanol, 1 h per concentration). Subsequently, the tissue blocks were rendered transparent using xylene and embedded in paraffin wax. The paraffin-embedded blocks were sectioned into slices with a thickness of 5 μm, which were then flattened in a warm water bath at 42 °C and mounted onto slides. The slices were baked in an oven at 45 °C for 30 min, followed by dewaxing in xylene and rehydration through a graded ethanol series. Finally, the sections were stained with hematoxylin and eosin.

### Transmission electron microscope

Fresh samples of pig liver tissue (1 mm^3^) were fixed in a solution of 2.5% glutaraldehyde and 2% paraformaldehyde for 24 h at 4 °C. The samples were subsequently rinsed three times with 0.1 mol/L phosphate buffer (pH 7.4), each for 10 min, before being fixed in 1% osmium tetroxide for 2 h in the dark. Following dehydration through a graded ethanol series (30%, 50%, 70%, 80%, 95%, and 100%), the specimens were treated with 100% acetone. Afterward, they were infiltrated and embedded with a mixture of acetone and 812 embedding agent. Polymerization was carried out for 48 h at 60 °C. The samples were sectioned into semi-thin sections (1.5 μm, stained with toluidine blue) and ultra-thin sections (60–80 nm). The ultra-thin sections mounted on formvar-coated grids were stained with 2% uranyl acetate (8 min in the dark) and 2.6% lead citrate (8 min under carbon dioxide-free conditions). The sections on copper grids were dried overnight at room temperature. The ultrastructure of the liver tissue was examined using transmission electron microscopy.

### RNA sequencing (RNA-seq)

Total RNA was isolated from piglet liver tissues using TRIzol® Reagent (Invitrogen, Carlsbad, CA, USA) following the manufacturer’s protocol. RNA quality and integrity were verified using a NanoDrop™ 2000 spectrophotometer and Agilent Bioanalyzer 2100 (Agilent Technologies, Santa Clara, CA, USA). Polyadenylated mRNA was enriched from total RNA (1 μg) using the PolyATtract® mRNA Isolation System III (Promega, Madison, WI, USA), which selectively binds poly(A) + RNA via magnetic oligo(dT) beads. Purified mRNA was fragmented into 200–300 bp fragments using Magnesium RNA Fragmentation Buffer (Thermo Fisher Scientific) at 94 °C for 5 min. Subsequently, cDNA was synthesized using fragmented mRNA as a template, amplified, and purified. Finally, the construction of the mRNA library was completed. After the library passed the quality inspection, it was sequenced on the Illumina HiSeq X-ten platform. Differential gene expression and enrichment analysis of sequencing data were conducted using bioinformatics tools. Genes with a −log_10_ (*P*-value) greater than 1 and a log_2_ fold change (log_2_FC) greater than 1 or less than −1 were considered significantly differentially expressed.

### Statistical analysis

The statistical package for the social sciences (SPSS) 26.0 was used for statistical analysis. GraphPad Prism 8.4.2 software was used for graphing. The experimental data were presented as mean ± standard error of the mean (SEM). Statistical comparisons between two groups were analyzed by two-tailed Student’s *t*-test, with significance levels indicated as follows: ^*^P < 0.05, ^**^P < 0.01, and ^***^P < 0.001. For the CCK-8 assay, one-way analysis of variance (ANOVA) was applied to compare differences among groups. 

## Results

### Iron deficiency disrupted hepatic iron metabolism and induced oxidative stress in suckling piglets

**Fig. 1:**
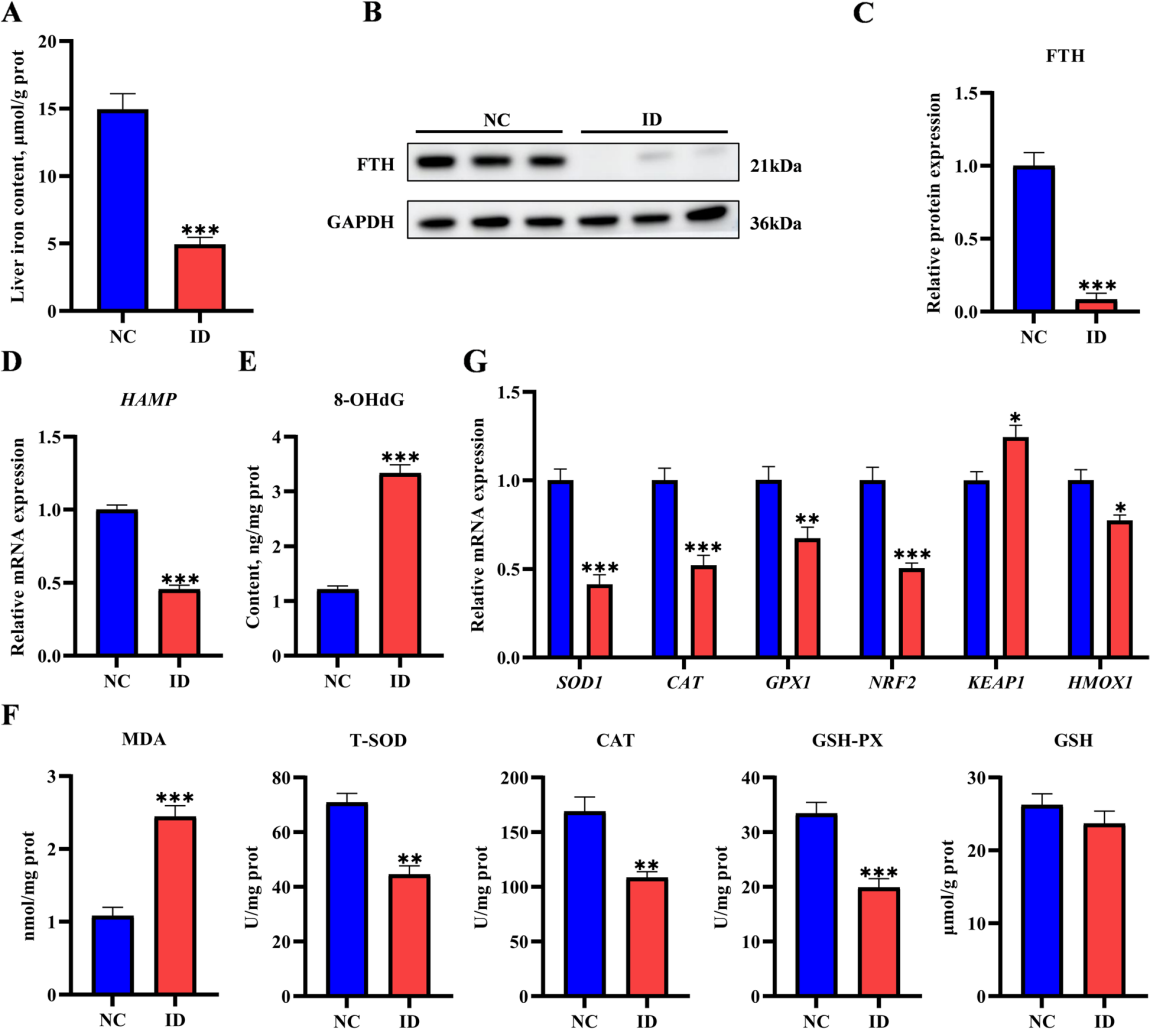
Iron deficiency disrupted hepatic iron metabolism and induced oxidative stress in suckling piglets. **A** Relative iron content in the NC and ID groups (*n* = 6). **B** and **C** Protein expression level of ferritin heavy chain (FTH) was detected by Western blot (*n* = 3). **D** Relative mRNA expression level of *HAMP* (encoding hepcidin) in the NC and ID groups (*n* = 6). **E** 8-OHdG levels in suckling piglets from the NC and ID groups (*n* = 6).** F** Hepatic MDA content, T-SOD activity, CAT activity, GSH-PX activity, and GSH content in the NC and ID groups (*n* = 6). **G** Relative mRNA expression levels of antioxidant and redox-related genes (*SOD1*, *CAT*, *GPX1*, *NRF2*, *KEAP1*, and *HMOX1*) in the NC and ID groups (*n* = 6). Statistical significance was determined using a two-tailed Student’s *t*-test and is marked as ^*^P < 0.05, ^**^P < 0.01, and ^***^P < 0.001

Compared with the NC group, hepatic iron content was significantly reduced in the ID group (Fig. 1A). Additionally, ferritin heavy chain (FTH), a core iron-storage protein, was markedly downregulated in the ID group (Fig. 1B and C). These results confirmed the successful generation of the iron-deficient piglet model. Consistent with this systemic iron deficiency, hepatic *HAMP* mRNA expression, which encodes the iron-regulatory hormone hepcidin, was significantly downregulated in the ID group (*P* < 0.001, Fig. 1D). To evaluate the impact of ID on hepatic oxidative injury, we measured oxidative stress markers and related gene expression profiles. The level of 8-OHdG, a specific biomarker of ROS-induced DNA damage [21], was significantly elevated in the livers of ID piglets (*P* < 0.001, Fig. 1E), indicating pronounced oxidative DNA damage. As shown in Fig. 1F, the activities of hepatic antioxidant enzymes were significantly inhibited in the ID group: T-SOD, CAT, and GSH-PX activities decreased by 37.1%, 35.7%, and 40.5% respectively (*P* < 0.01), while the content of MDA, the end product of lipid peroxidation, significantly increased by 2.7 times (*P* < 0.001), with no effect on the GSH levels. Additionally, ID significantly downregulated the mRNA expression of oxidative stress-related genes, including nuclear factor E2-related factor 2 (*NRF2*), *SOD1*, *CAT*, *GPX1*, and heme oxygenase 1(*HMOX1*, encodes the HO-1 protein), while upregulating the mRNA expression of Kelch-like ECH-associated protein 1 (*KEAP1*; Fig. 1G). These results indicated that ID disrupted the antioxidant defense system Nrf2-Keap1, leading to redox imbalance and oxidative liver injury in suckling piglets.

### Iron deficiency induced endoplasmic reticulum stress via UPR activation in suckling piglet liver

**Fig. 2:**
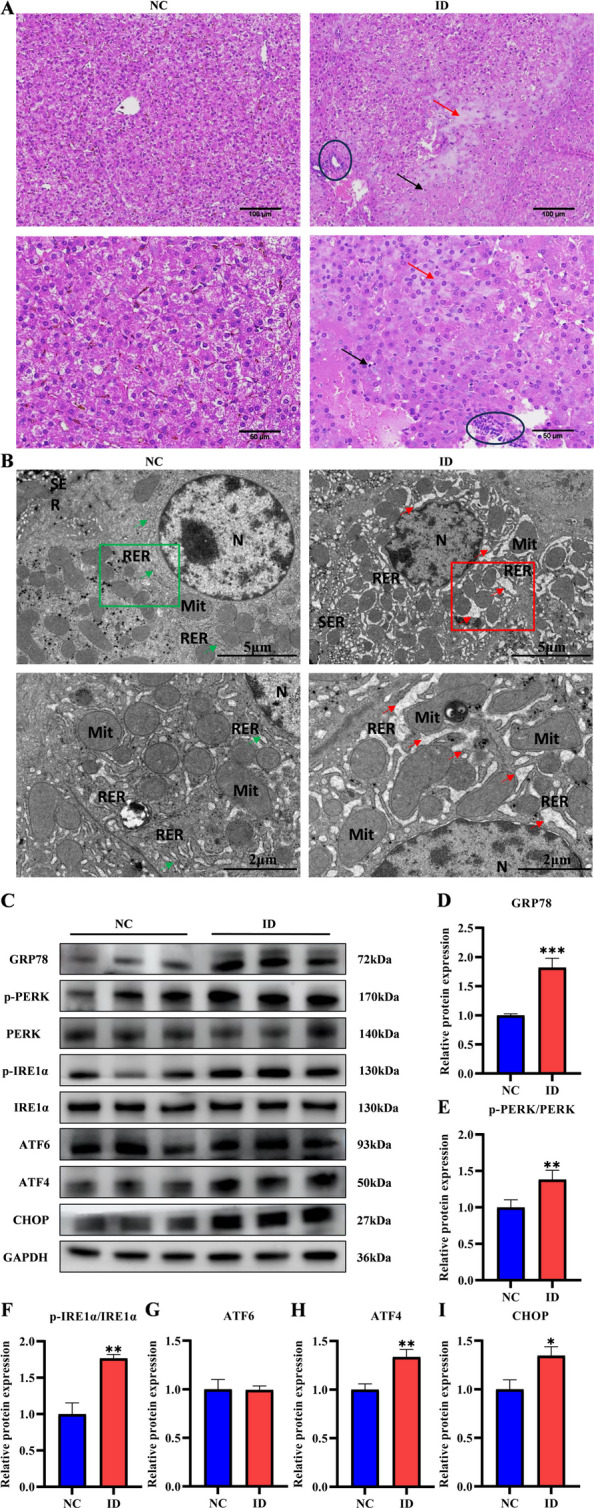
Iron deficiency induced ERS via UPR activation in suckling piglet liver. **A** H&E staining of suckling piglet livers in the NC and ID groups (*n* = 3). Scale bar, 50 μm, 100 μm. **B** TEM of suckling piglet livers in the NC and ID groups (*n* = 3). Scale bar, 2 μm, 5 μm. **C**–**I** Western blot analysis of the protein expression of ERS markers and UPR signaling pathways in the NC and ID groups (*n* = 3). Statistical significance was determined using a two-tailed Student’s *t*-test and is marked as ^*^P < 0.05, ^**^P < 0.01, and ^***^P < 0.001

Histopathological analysis revealed distinct morphological differences between the groups. Meanwhile, the NC group displayed intact hepatocyte cytoarchitecture with organized hepatic cords, defined sinusoids, homogeneous eosinophilic cytoplasm, and regular nuclei. In contrast, the ID group exhibited disorganized cords, cytoplasmic vacuolation, heterogeneous cellular density, and focal pale eosinophilic staining (Fig. 2A). These histopathological outcomes suggest hydropic degeneration and mononuclear inflammatory cell infiltration, as well as inflammation and significant hepatic parenchymal damage. The ultrastructural analysis by TEM also confirmed these differences. The NC group hepatocytes had preserved subcellular architecture: ovoid nuclei with intact membranes and nuclear pores, mitochondria with intact cristae, and organized parallel RER cisternae with tight ribosome adherence adjacent to perinuclear/mitochondrial regions. While the ID group hepatocytes showed pronounced derangements: irregular nuclear contours with membrane invagination, and RER remodeling featuring vesiculated cisternae with luminal dilation and ribosomal detachment (Fig. 2B). Since RER integrity is essential for protein folding, its structural disruption in ID piglets likely impairs folding capacity, leading to the accumulation of misfolded proteins and triggering the UPR. Consistent with this hypothesis, the protein levels of the ER chaperone GRP78 were significantly upregulated in the ID group (Fig. 2C–I), as the phosphorylation levels of UPR sensors PERK (Thr980) and IRE1α (Ser724) significantly increased, while total ATF6 expression remained unchanged, indicating UPR activation primarily through the PERK and IRE1α branches. The protein levels of the pro-apoptotic transcription factor ATF4 and its downstream target CHOP were also significantly elevated, confirming that ERS drives hepatocyte apoptosis via the ATF4-CHOP axis.

### Iron deficiency induced hepatic inflammation and activated TLR4/NF-κB pathway in suckling piglets

**Fig. 3:**
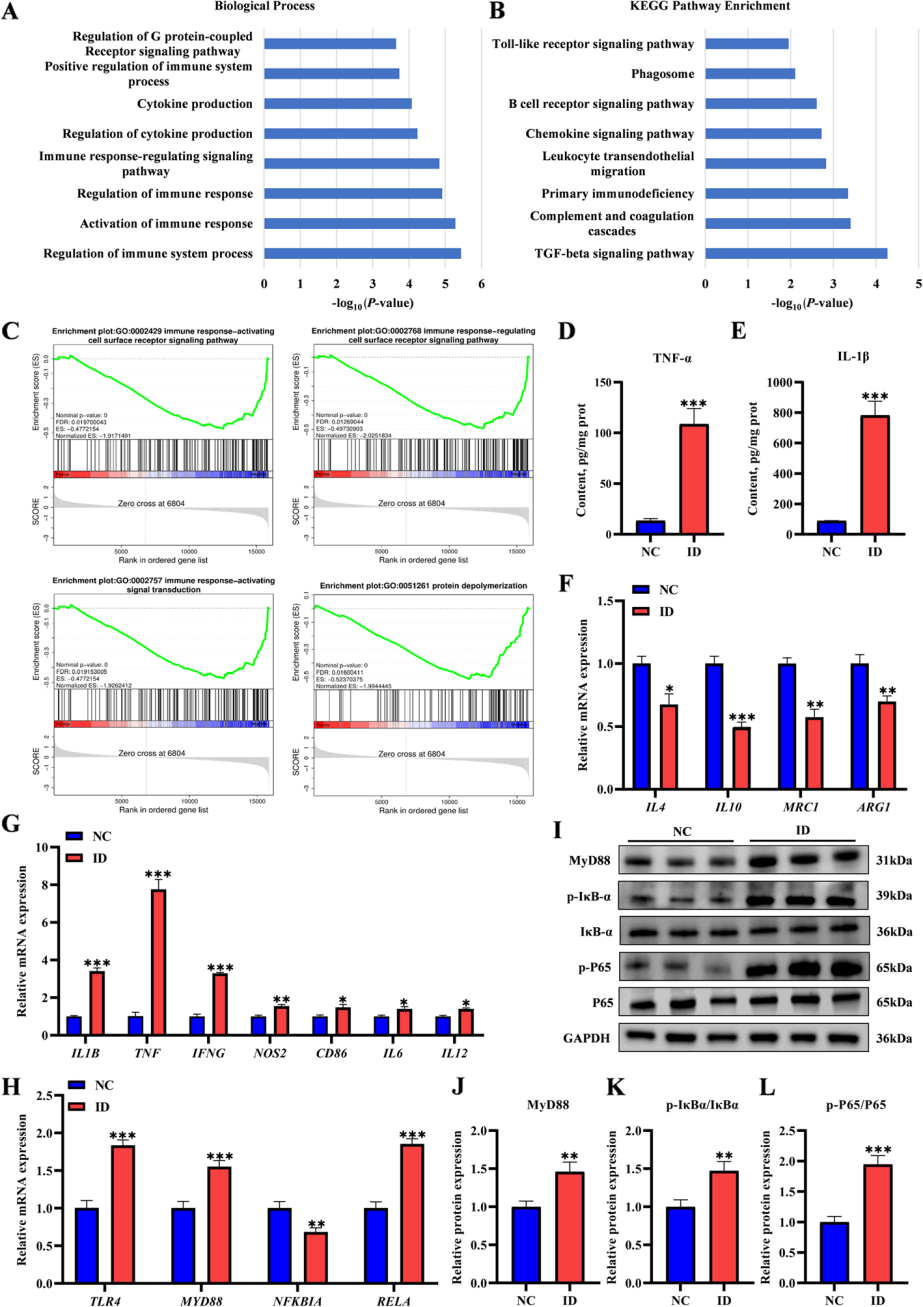
Iron deficiency induced hepatic inflammation and activated TLR4/NF-κB pathway in suckling piglets. **A** and **B** Gene Ontology (GO) and Kyoto Encyclopedia of Genes and Genomes (KEGG) enrichment analysis of differentially expressed genes of suckling piglet livers, focusing on the biological process and signaling pathway (*n* = 3). **C** Gene set enrichment analysis (GSEA) between the NC and ID groups. **D** and **E** ELISA detection of IL-1β and TNF-α contents in the NC and ID groups (*n* = 6). **F** and **G** qRT-PCR analysis of expression of M2-type macrophage markers, anti-inflammatory cytokines, M1-type macrophage markers and pro-inflammatory cytokines in the NC and ID groups (*n* = 6). **H** qRT-PCR analysis of the gene expression of TLR4/NF-κB signaling pathway in the NC and ID groups (*n* = 6). **I**–**L** Western blot analysis of the protein expression of TLR4/NF-κB signaling pathway in the NC and ID groups (*n* = 6). Statistical significance was determine using a two-tailed Student’s *t*-test and is marked as ^*^P < 0.05, ^**^P < 0.01, and ^***^P < 0.001. NES: Normalized enrichment score; FDR: False discovery rates

RNA-seq profiling of liver tissue from suckling piglets demonstrated a significant enrichment of immune-related terms (biological processes GO and KEGG pathways) in the ID group, such as activation of immune responses, regulation of immune responses, cytokine production, chemokine signaling pathway, and Toll-like receptor (TLR) signaling pathway (Fig. 3A and B). These findings suggest that ID may dysregulate hepatic immune-inflammatory responses. Gene set enrichment analysis (GSEA) further supported these findings, highlighting alterations in immune response-associated signal transduction and protein depolymerization (Fig. 3C). In line with the transcriptomic data, the hepatic contents of IL-1β and TNF-α were significantly increased in the ID group compared to the NC group (Fig. 3D and E). The mRNA expression levels of M2-type macrophage markers [mannose receptor C-type 1 (*MRC1*), arginase 1 (*ARG1*)] and anti-inflammatory cytokines (*IL4* and *IL10*) were significantly downregulated (Fig. 3F). Meanwhile, the mRNA expression levels of M1-type macrophage markers [nitric oxide synthase 2 (*NOS2*), and CD86 molecule (*CD86*)] and pro-inflammatory cytokines [*IL1B*, *TNF*, interferon gamma (*IFNG*), interleukin 6 (*IL6*), and interleukin 12 (*IL12*)] were significantly upregulated in the ID group (Fig. 3G). These results collectively indicated that ID disrupted the balance between pro- and anti-inflammatory responses in the liver, polarizing the local immune microenvironment toward a pro-inflammatory state. Furthermore, the mRNA expression levels of Toll-like receptor 4 (*TLR4*), myeloid differentiation primary response 88 (*MYD88*), and RELA proto-oncogene (*RELA*, encoding the NF-κB p65 subunit) were significantly increased in the livers of the ID group, while the mRNA expression level of NFKB inhibitor alpha (*NFKBIA*) was significantly decreased (Fig. 3H). At the protein level, the expression of MYD88 and the phosphorylation ratios of NF-κB p65 (p-NF-κB p65/NF-κB p65) and IκBα (p-IκBα/IκBα) were significantly increased (Fig. 3I–L). These results collectively indicated that ID promoted hepatic inflammatory injury in suckling piglets through activation of the TLR4/NF-κB signaling axis.

### Iron deficiency induced inflammation and ERS in hepatocytes

**Fig. 4:**
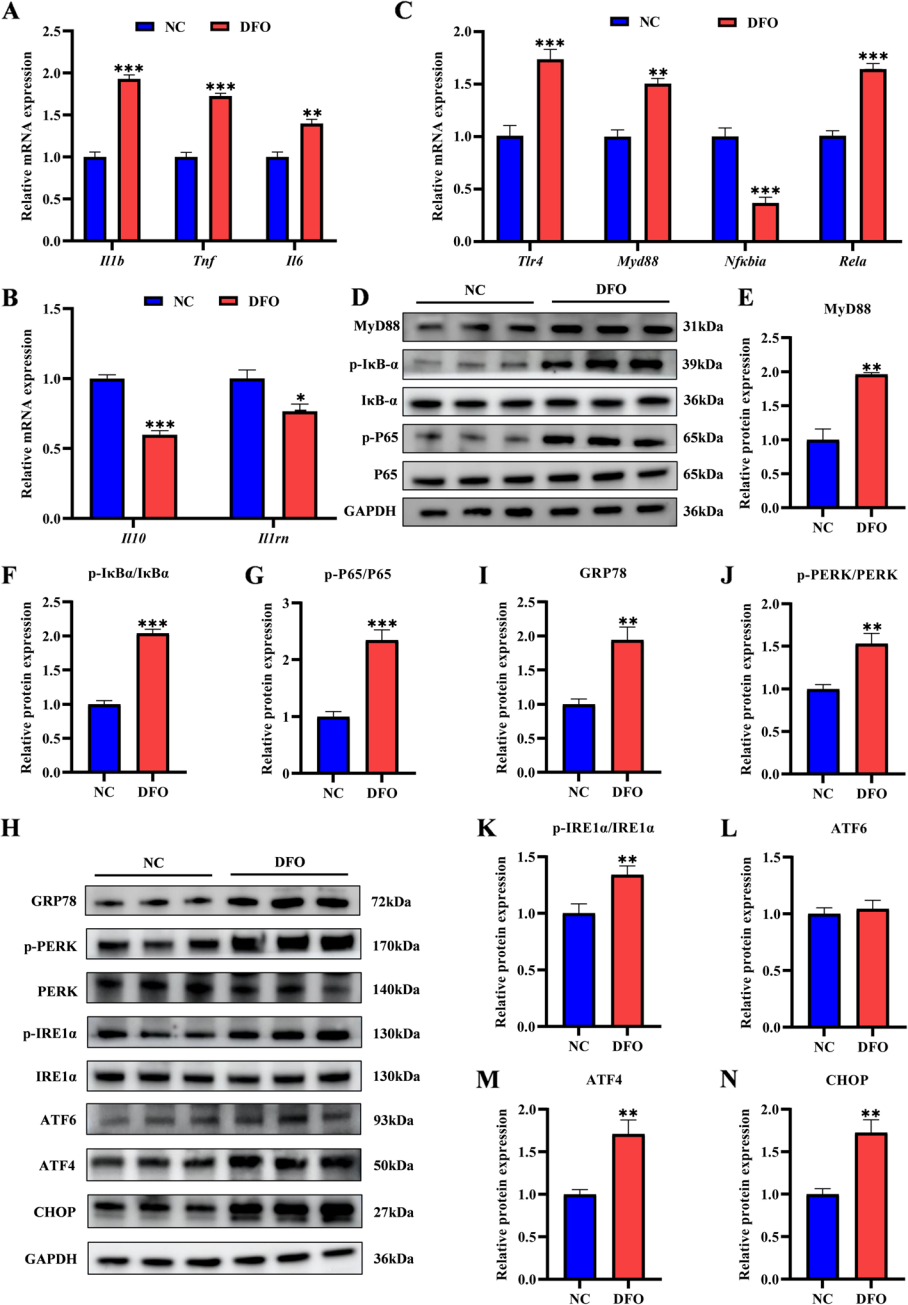
Iron deficiency induced inflammation and ERS (Endoplasmic reticulum stress) in hepatocytes (*n* = 3). **A** and **B** qRT-PCR analysis of expression of pro-inflammatory and anti-inflammatory related genes. **C** qRT-PCR analysis of the gene expression of TLR4/NF-κB signaling pathway. **D**–**G** Western blot analysis of the protein expression of TLR4/NF-κB signaling pathway. **H**–**N** Western blot analysis of the protein expression of ERS markers and UPR signaling pathways in AML12 cells. Statistical significance was determined using a two-tailed Student’s *t*-test and is marked as ^*^P < 0.05, ^**^P < 0.01, and ^***^P < 0.001

DFO has been widely applied to induce iron-deficient conditions in both cellular and animal models [22, 23], depleting bioavailable iron by forming stable complexes with Fe^3+^ [24]. To model iron deficiency (ID) in vitro, AML12 cells were treated with 300 μmol/L DFO. Under this condition, ID significantly upregulated the expression of pro-inflammatory genes (*Il1b*, *Tnf*, and *Il6*) (Fig. 4A), while downregulating anti-inflammatory genes *Il10* and interleukin-1 receptor antagonist (*Il1rn*) in AML12 hepatocytes (Fig. 4B). Furthermore, the mRNA expression levels of *Tlr4*, *Myd88*, and *Rela* were significantly increased in the DFO group, whereas the expression of *Nfkbia* was decreased (Fig. 4C). Concordantly, protein analysis revealed elevated MYD88 expression and enhanced phosphorylation of both NF-κB p65 and IκBα, confirming robust activation of the TLR4/NF-κB signaling axis under ID conditions (Fig. 4D–G). In addition, ERS was also induced by DFO treatment, as indicated by the upregulation of UPR-related proteins GRP78, ATF4, and CHOP, together with increased phosphorylation of PERK and IRE1α (Fig. 4H–N). These in vitro results aligned closely with our in vivo observations, collectively establishing ID as a potent inducer of both ERS and inflammatory signaling in hepatocytes. Notably, IL-1β and TNF-α, as core inflammatory mediators, were consistently upregulated across both models in response to ID.

### ERS induced inflammatory responses and NF-κB pathway

**Fig. 5:**
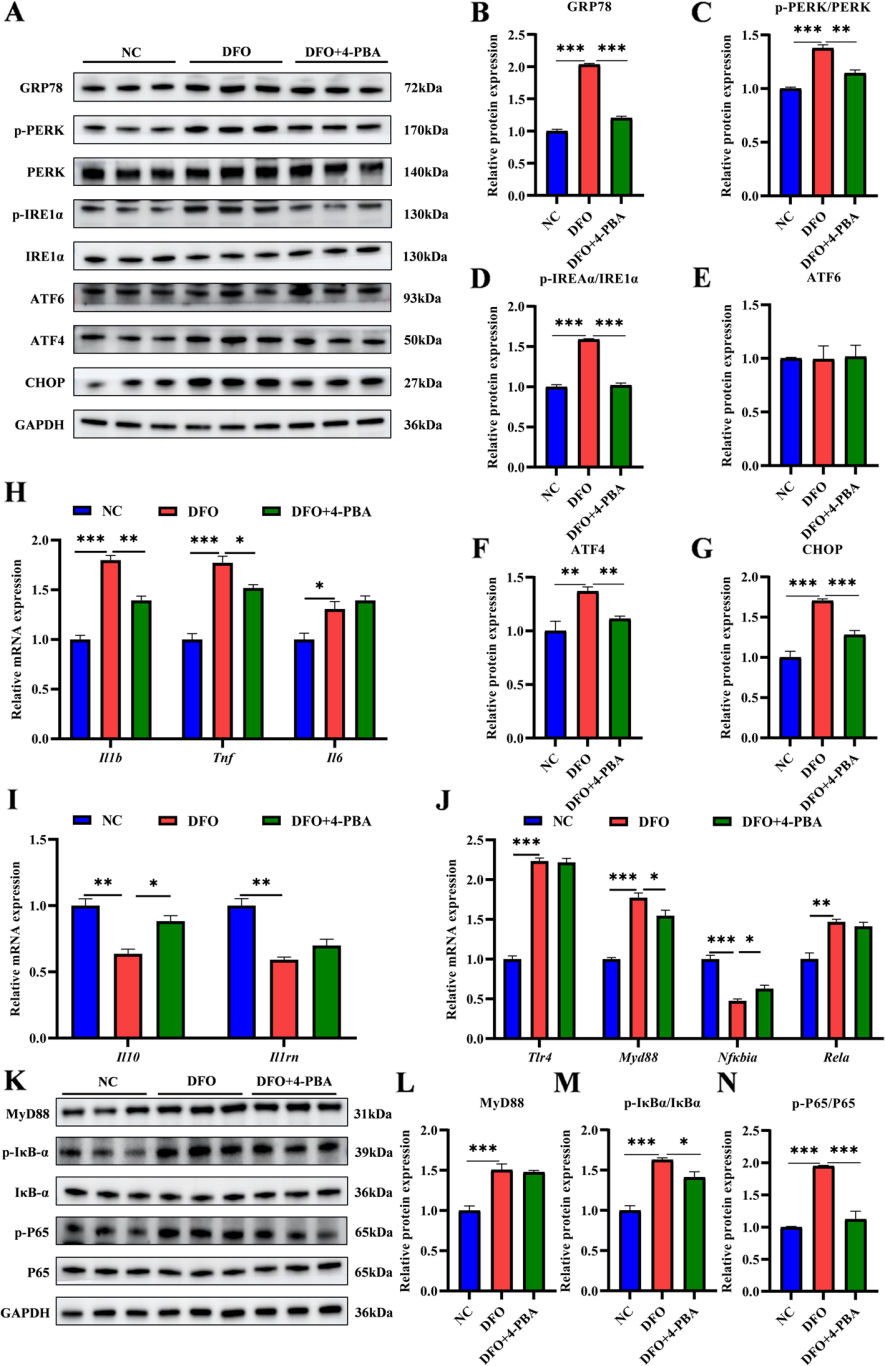
ERS induced inflammatory responses and NF-κB pathway. **A**–**G** Western blot analysis of the protein expression of ERS markers and UPR signaling pathways in the NC, DFO and 4-PBA supplement groups (*n* = 3). **H** and **I** qRT-PCR analysis of expression of pro-inflammatory and anti-inflammatory related genes (*n* = 3). **J**–**N** Analysis of TLR4/NF-κB signaling pathway expression by qRT-PCR and Western blot (*n* = 3). Statistical significance was determined using a two-tailed Student’s *t*-test and is marked as ^*^P < 0.05, ^**^P < 0.01, and ^***^P < 0.001

Endoplasmic reticulum stress (ERS) is a recognized upstream trigger of inflammatory responses, yet its direct causal role in hepatic inflammation during iron deficiency (ID) in suckling piglets remained unclear. To address this, we examined the effect of specific ERS inhibition in an in vitro ID model using AML12 hepatocytes. Co-treatment of DFO (300 μmol/L) with the chemical chaperone 4-PBA (2 mmol/L), which suppresses UPR activation [25], showed low cytotoxicity (86% cell viability, Fig. S1A) and significantly reduced protein levels of ERS markers, including GRP78, phospho-PERK, ATF4 and phospho-IRE1α, with no effect on ATF6 expression (Fig. 5A–G). 4-PBA pretreatment mitigated the pro-inflammatory effects of ID, downregulating the mRNA expression of pro-inflammatory cytokines (*Il1b* and *Tnf*) and upregulating the anti-inflammatory cytokine *Il10* (Fig. 5H and I). Moreover, 4-PBA-mediated ERS inhibition attenuated ID-induced hyperactivation of the NF-κB pathway (mRNA expression of *Myd88* and *Nfkbia*), and significantly reduced phosphorylation levels of IκBα and NF-κB p65 proteins (Fig. 5J–N). Collectively, pharmacological inhibition of ERS by 4-PBA significantly attenuated ID-induced inflammation in AML12 hepatocytes. These data mechanistically indicated that ERS serves as an essential upstream driver of hepatocellular inflammation under iron-deprived conditions, wherein the NF-κB signaling cascade acts as a critical downstream executor.

## Discussion

Newborn piglets are highly susceptible to ID due to rapid growth, low innate iron reserves, and the insufficient iron content in sow milk. Although intramuscular dextran iron injection (150–200 mg on the third day of age) is widely used, up to 43% of piglets still exhibit ID after weaning. This underscores its persistent challenge in pig production [26]. Previous studies have largely focused on hematological and growth performance implications [27, 28]. However, the molecular mechanisms underlying ID-induced hepatic injury, particularly inflammation, remain poorly understood. Our results revealed that ID initiated a vicious cycle of hepatic damage through oxidative stress-triggered ERS. Specifically, ID impaired electron transport chain (ETC) function and iron-dependent antioxidant enzymes, leading to ROS accumulation (quantified by elevated hepatic 8-OHdG) and suppression of the Nrf2 pathway. This oxidative burden activated the PERK and IRE1α branches of the unfolded protein response, which in turn promoted NF-κB-mediated transcription of pro-inflammatory cytokines such as IL-1β and TNF-α. These findings establish a mechanistic basis for ID-induced liver inflammation and provide a theoretical foundation for targeted nutritional interventions.

The dynamic balance between the oxidative and antioxidant systems is fundamental to maintaining health. The antioxidant system in newborn piglets is not fully mature at birth, making them particularly vulnerable to oxidative damage during the first postnatal week, which is gradually restored as the system matures [29]. Oxidative stress is a common pathological feature of chronic diseases associated with prolonged ID [30–32]. Iron, as an essential component of key ETC cofactors including iron-sulfur clusters (ISCs) and heme (e.g., in cytochromes), is critical for ETC function [33]. Iron deficiency disrupts ISC synthesis and compromises cytochrome function, thereby reducing electron transfer efficiency in the ETC and increasing ROS generation [33]. Previous studies have confirmed that maternal ID during pregnancy causes fetal mitochondrial dysfunction and oxidative stress in rodents [34]. Furthermore, patients with ID anemia exhibit elevated blood ROS levels and reduced antioxidant enzyme activity [35], while ID exacerbates hepatic lipid peroxidation in rats [36]. Consistent with these findings, our study demonstrated that suckling piglets with iron deficiency exhibited significantly elevated hepatic 8-OHdG levels (a gold-standard surrogate marker for ROS-mediated DNA oxidative damage) alongside significantly downregulated activity of key antioxidant enzymes, including SOD, CAT, and GPX. This impairment of antioxidant defense capacity diminished systemic ROS clearance efficiency, inducing marked oxidative stress manifested not only by the excessive accumulation of 8-OHdG but also by increased MDA, a lipid peroxidation product. Importantly, MDA decomposition generates additional free radicals, which further promote 8-OHdG formation by enhancing ROS-mediated DNA oxidation, establishing a vicious cycle that further exacerbates hepatic oxidative damage [37].

The transcription factor Nrf2 serves as a master regulator of cellular antioxidant defense, maintaining redox homeostasis by activating the expression of genes encoding antioxidant enzymes (e.g., SOD, CAT, GSH-PX) and detoxifying enzymes [38]. Under physiological conditions, its negative regulator, Keap1, promotes the ubiquitin-dependent degradation of Nrf2 [39]. However, under oxidative stress, conformational changes in Keap1 lead to the release of Nrf2, enabling its nuclear translocation and subsequent activation of downstream target gene transcription, including *HMOX1*, thereby restoring cellular homeostasis [39]. HO-1 (encoded by the *HMOX1* gene) possesses multifaceted functions that encompass antioxidant and anti-inflammatory activities, as well as involvement in regulating iron metabolism [40]. Previous studies have demonstrated that prolonged ID reduces Nrf2 protein expression and decreases autophagic flux in the mouse hepatocyte cell line AML12 [17]. Consistent with these findings, our study revealed that suckling piglets with iron deficiency exhibited significantly decreased hepatic mRNA expression levels of NRF2 and its critical target gene *HMOX1*, concomitant with significantly increased mRNA expression of *KEAP1*. This pattern suggests that ID suppresses the activation of the Nrf2 pathway. The transcriptional upregulation of *KEAP1* likely enhances the suppression of the Nrf2 protein, resulting in reduced expression of downstream antioxidant genes, such as *HMOX1*. This functional impairment of the Nrf2/Keap1-HO-1 signaling axis significantly compromises the hepatic antioxidant capacity in suckling piglets, thereby exacerbating oxidative stress-induced damage.

The liver functions as the principal organ for metabolic detoxification and immune regulation. It maintains a dynamic equilibrium between immune tolerance and defense through its specialized cellular composition and intricate signaling networks. Central to this immune competency is the coordinated interaction among immune cells and the precise balance between pro- and anti-inflammatory cytokines in hepatocytes.

Iron is an essential cofactor for numerous immunologically active molecules and plays a critical role in hepatic immunity, which is indispensable for the synthesis of hemoglobin and immunoglobulins [41]. Critically, ID impairs mRNA translational efficiency in hepatocytes, leading to significantly reduced hepatic globulin and total protein production. This directly inhibits complement system function and antibody generation, decreasing both humoral and cellular immunological responses [10, 42]. In piglet models, this mechanism manifests as suppressed lactoferrin synthesis, which diminishes its inhibitory capacity against intestinal pathogens and contributes to the high incidence of diarrhea [43]. Studies in murine models further indicate that ID promotes a systemic pro-inflammatory state, characterized by elevated leukocyte counts, upregulated transcription of hepatic acute-phase proteins, and enhanced infiltration of inflammatory cells into the liver [44]. Additionally, ID increases susceptibility to infections by disrupting macrophage bactericidal activity and impairing T-cell proliferation and function [45]. Prior studies have reported significantly elevated serum levels of pro-inflammatory cytokines (IL-1β, IL-4, IL-6, TGF-β1, and TNF-α) and rising trends in IL-12 and granulocyte–macrophage colony-stimulating factor (GM-CSF) in iron-deficient weaned piglets [46]. Among these cytokines, IL-1β and TNF-α act as key inflammatory mediators that drive chronic inflammation during hyperactivated immune responses. In the present study, we detected significant upregulation of pro-inflammatory factors (*IL1B*, *TNF*, *IFNG*, *NOS2*, *CD86*, *IL6* and *IL12*) along with downregulation of anti-inflammatory mediators (*IL4*, *IL10*, *MRC1* and *ARG1*) in the livers of iron-deficient suckling piglets, confirming that ID skews hepatic immunity toward a pro-inflammatory state and causes liver injury. Interferon-γ (IFN-γ), a central regulator of macrophage iron status and function, stimulates nitric oxide (NO) production via inducible nitric oxide synthase (iNOS) mediating innate immune responses [47]. The expression of iNOS, the key enzyme responsible for NO generation, is under complex iron-dependent control. Experiments involving iron chelators and iron supplementation consistently show an inverse relationship between cellular iron status and iNOS expression [48]. Previous in vivo studies confirmed that high-iron diets suppress IFN-γ expression [49]. In line with these findings, our data indicated that ID significantly upregulated hepatic *IFNG* and *NOS2* mRNA levels in suckling piglets.

Hepcidin, encoded by the *HAMP* gene, serves as a master regulator of systemic iron metabolism by inhibiting intestinal iron absorption and macrophage iron recycling [50]. Although hepatic inflammation typically induces hepcidin upregulation [51], we observed significant suppression of *HAMP* in ID piglets, indicating that the ID signal overrides inflammatory stimuli. This regulation of IDA is mechanistically mediated through the BMP6-SMAD signaling pathway, which transcriptionally represses HAMP as an adaptive strategy to enhance iron absorption and mobilization [50]. Although inflammation driven by ID-mediated ERS/NF-κB activation has the potential to promote *HAMP* expression, its effect is superseded by the dominant inhibitory signal of iron deficiency. Consequently, reduced hepcidin levels facilitate dietary iron uptake in the duodenum and promote iron efflux from macrophages, thereby increasing iron availability for erythropoiesis and contributing to correct anemia.

The iron chelator DFO was used to establish an in vitro model of ID in AML12 cells. DFO depletes intracellular and extracellular iron pools by forming stable complexes with ferric ions (Fe^3+^) [22]. In this model, ID significantly upregulated the mRNA expression of pro-inflammatory cytokines, including *Il1b*, *Tnf*, and *Il6*, while concurrently downregulating anti-inflammatory mediators such as *Il10* and *Il1rn*. This dysregulated cytokine profile confirmed that ID directly induced an inflammatory response in AML12 cells.

TLR4, as a critical innate immune sensor, specifically recognizes conserved molecular motifs, including pathogen-associated molecular patterns (PAMPs) and endogenous damage-associated molecular patterns (DAMPs) [52]. Upon activation, TLR4 recruits downstream signaling molecules via the MyD88-dependent pathway, leading to NF-κB pathway activation [53]. NF-κB, a master transcriptional regulator of inflammation and immune responses (with the RelA/p65 subunit being functionally predominant), resides in the cytoplasm as an inactive complex bound to its inhibitor IκB under resting conditions [54]. TLR4 ligation triggers MyD88-mediated phosphorylation of the IκB kinase (IKK) complex, which phosphorylates IκBα, thereby inducing its ubiquitination and degradation. This process liberates the p65 subunit, enabling its phosphorylation (p-p65) and nuclear translocation to drive pro-inflammatory cytokine gene expression [55]. Our study demonstrated significant activation of the TLR4/NF-κB axis in ID suckling piglets and AML12 hepatocytes, evidenced by the coordinated upregulation of MyD88, p-IκBα/IκBα, and p-p65/p65 protein levels. These findings established that ID triggered hepatic inflammatory injury through TLR4/NF-κB signaling. It has been reported that during hepatic ischemia–reperfusion injury, TLR4 activation via NF-κB ignites a cytokine storm that amplifies tissue damage [56]. Furthermore, the genetic ablation of *Tlr4* abolished hepatic inflammation and injury of methionine-choline deficient (MCD) diet-induced nonalcoholic steatohepatitis (NASH) mice, underscoring TLR4's central role in inflammatory cascades [57].

The ER serves as a critical cellular organelle essential for protein synthesis, post-translational modification, folding, and the maintenance of intracellular calcium ion homeostasis [16]. Redox environment and iron-dependent enzymes, such as prolyl hydroxylase and cysteine dioxygenase, constitute a necessary condition for the formation of protein disulfide bonds and correct spatial folding [58]. Under normal conditions, the ER molecular chaperone GRP78 binds to membrane proteins to maintain ER homeostasis [16]. However, upon energy disturbance or oxidative stress induced by external environmental changes, the aberrant accumulation of unfolded or misfolded proteins triggers ERS, activating the UPR to restore homeostasis. The UPR is initiated by the transmembrane proteins (IRE1, PERK, and ATF6) [16]. Iron, as a key component of the mitochondrial oxidative respiratory chain, deficiency leads to a significant increase in mitochondrial ROS levels [33]. As previously reported, the SOD2 upregulation in cardiomyocytes and neuroblastoma cells, induces the antioxidant system, moderating ERS [18, 19]. Our study observed significantly elevated phosphorylation levels of PERK and IRE1α in the livers of ID suckling piglets and in DFO-treated AML12 cells, while ATF6 expression showed no significant changes. These findings indicated that the PERK and IRE1α pathways play dominant roles in ID-induced ERS. ATF4 is activated early during ERS and aids cellular recovery of homeostasis by regulating amino acid metabolism, antioxidant genes, and autophagy-related genes [59]. Under sustained stress, ATF4 initiates the transcription of the pro-apoptotic factor CHOP, shifting cellular fate towards apoptosis [59]. The current study further demonstrated significantly increased protein expression of both ATF4 and CHOP in the livers of iron-deficient piglets, suggesting that prolonged ID induces severe ERS. Mechanistically, ID likely impairs mitochondrial oxidative phosphorylation by disrupting iron-sulfur cluster biosynthesis and cytochrome function, reducing electron transport chain efficiency and promoting excessive ROS generation. The downregulated activity of the hepatic antioxidant enzyme system also reduces ROS scavenging capacity. These combined effects lead to the abnormal accumulation of unfolded or misfolded proteins, ultimately inducing ERS. The accumulation of unfolded or misfolded proteins within the ER lumen induces excessive ROS generation, establishing a vicious cycle between oxidative stress and ERS [58].

ERS is a well-established trigger of inflammatory responses and is implicated in the pathogenesis of various chronic inflammatory diseases, including diabetes, obesity, neurodegenerative disorders, and atherosclerosis [60–62]. At the molecular level, immunoglobulin μ heavy chain overexpression can activate NF-κB, a complex typically composed of p50/p65 or p50/c-Rel heterodimers, through ER-to-nucleus signaling pathways, leading to ER protein accumulation [63]. Furthermore, all three branches of the UPR contribute to NF-κB signaling [64]. Knockdown studies have demonstrated that both IRE1α and PERK are required for full NF-κB activation within the ERS pathway, indicating their synergistic roles [65]. Specifically, the PERK-eIF2α-ATF4 axis upregulates CHOP, which in turn promotes NF-κB-mediated inflammatory amplification [66]. Activated IRE1α recruits tumor necrosis factor receptor-associated factor 2 (TRAF2), leading to NF-κB activation through complexes such as IKK [67]. The ATF6 branch can stimulate the expression of inflammatory mediators via AKT-NF-κB signaling and the ATF6-XBP1-CHOP pathway [68, 69]. In the present study, the ERS inhibitor 4-PBA, by stabilizing the ER protein folding environment, significantly downregulated the phosphorylation levels of IκB and p65 in iron-deficient AML12 cells, thereby inhibiting the NF-κB signaling pathway and alleviating the inflammatory response. These results supported a mediating role for ERS in iron deficiency-induced hepatic inflammation. ROS are highly reactive molecules with unpaired electrons that also serve as key inflammation triggers [70]. The accumulation of misfolded proteins in the ER can provoke calcium leakage [71]. This calcium accumulates in the mitochondrial matrix, causing inner membrane depolarization, disrupting electron transport, and increasing ROS generation. Calcium dysregulation, oxidative stress, and proteostatic failure interact to activate NF-κB, increase inflammation, and potentially cause cell death [71]. The interaction between inflammation and ERS is bidirectional, as inflammatory cytokines can also induce ERS [72]. For instance, TNF-α activates PERK, IRE1α, and ATF6 in fibrosarcoma cells [73]. TNF-α, IL-1β, and IL-6 can induce ERS in hepatocytes and activate CREBH-mediated acute phase responses [74]. T cell-derived IFN-γ is associated with PERK activation and ERS-induced apoptosis in oligodendrocytes [75]. The underlying mechanism of IDA likely involves cytokines triggering ER calcium release and ROS accumulation, thereby interfering with protein folding and mitochondrial metabolism.

A key limitation of this study is that, although our models robustly show iron deficiency activates the oxidative stress-ERS-inflammatory axis, we could not identify the precise iron-sensing molecules or primary ER-resident enzymes responsible for UPR initiation. Consequently, future work using organoids or tissue-specific knockouts is warranted to dissect these cell-autonomous effects and define the key effector proteins within the ER.

## Conclusions

In conclusion, these findings demonstrated that ID triggers liver injury through a series of interconnected events: ID directly impaired ETC function, leading to excessive ROS accumulation. The ID state downregulated the activity of iron-dependent antioxidant enzymes, which compromising the liver's ability to scavenge ROS. Furthermore, ID suppressed the Nrf2 signaling pathway, exacerbating oxidative stress. These accumulated ROS activated the UPR pathways mediated by PERK and IRE1α. The PERK/IRE1α axis activated the NF-κB signaling pathway, triggering the release of pro-inflammatory cytokines such as IL-1β and TNF-α. This cascade established a self-amplifying vicious cycle of “oxidative stress-ERS-inflammation”, aggravating hepatic damage. Overall, this study provided an important theoretical basis for developing targeted interventions to treat ID-induced liver injury in suckling piglets.

## Supplementary Information

Additional file 1: Table S1. Ingredients composition of iron-deficient milk powder for suckling piglets. Table S2. Milk feeding schedule for newborn piglets. Table S3. Primer sequences and accession numbers of genes used in qRT-PCR(pig). Table S4. Primer sequences and accession numbers of genes used in qRT-PCR(mouse). Table S5. Details of antibodies. Fig. S1A. Cell viability was detected by CCK-8 assay in AML12 cells treated with 4-PBA and DFO.

Additional file 2. Original Western blot images.

## Data Availability

The RNA-seq data have been deposited in the NCBI Gene Expression Omnibus (GEO) under accession number GSE304597. The datasets supporting the conclusions of this article are included within the article and its additional file, further inquiries can be directed to the corresponding author.

## Abbreviations

4-PBA: 4-Phenylbutyric acid
8-OHdG: 8-Hydroxy-2'-deoxyguanosine
ARG1: Arginase 1
ATF4: Activating transcription factor 4
ATF6: Activating transcription factor 6
CD86: Cluster of differentiation 86
CHOP: C/EBP homologous protein
DFO: Deferoxamine
ERS: Endoplasmic reticulum stress
FTH: Ferritin heavy chain
GRP78: Glucose-regulated protein 78
HMOX1: Heme oxygenase 1
H&E: Hematoxylin and eosin
ID: Iron deficiency
IFNG: Interferon gamma
IL1B: Interleukin 1 beta
IRE1α: Inositol-requiring enzyme 1 alpha
Keap1: Kelch-like ECH-associated protein 1
MRC1: Mannose receptor C-type 1
MYD88: Myeloid differentiation primary response 88
NF-κB: Nuclear factor kappa-B
NFKBIA: NFKB inhibitor alpha
NOS2: Nitric oxide synthase 2
Nrf2: Nuclear factor E2-related factor 2
PERK: Protein kinase R-like endoplasmic reticulum kinase
RELA: RELA proto-oncogene
ROS: Reactive oxygen species
TEM: Transmission electron microscopy
TNF: Tumor necrosis factor
TLR4: Toll-like receptor 4
UPR: Unfolded protein response
