# Supplementary material for: Iron deficiency aggravates hepatic inflammation in suckling piglets via endoplasmic reticulum stress-driven NF-κB pathway activation
Source: J Anim Sci Biotechnol. 2026 Feb 13;17:30. doi: 10.1186/s40104-026-01356-4 (PMC12903631; doi:10.1186/s40104-026-01356-4)
Supplement: Supplementary file 1 — Additional file 1: Table S1. Ingredients composition of iron-deficient milk powder for suckling piglets. Table S2. Milk feeding schedule for newborn piglets. Table S3. Primer sequences and accession numbers of genes used in qRT-PCR(pig). Table S4. Primer sequences and accession numbers of genes used in qRT-PCR(mouse). Table S5. Details of antibodies. Fig. S1A. Cell viability was detected by CCK-8 assay in AML12 cells treated with 4-PBA and DFO. [file 40104_2026_1356_MOESM1_ESM.docx]

**Table S1.** Ingredients composition of iron-deficient milk powder for suckling piglets.

| Ingredient | Content (g/kg) |
| --- | --- |
| Skim milk powder | 160 |
| Whey powder | 426 |
| Whey protein concentrate | 190 |
| Soy lecithin powder | 150 |
| Compound vitamins | 3 |
| Corn starch | 18.3 |
| Dicalcium phosphate | 40 |
| Sodium selenite | 0.011827 |
| Manganese sulfate | 0.02375 |
| Copper sulfate | 0.016 |
| Zinc sulfate | 0.05246 |
| Calcium iodate | 0.027 |
| Magnesium sulfate | 0.137 |
| Sodium chloride | 10 |
| Potassium chloride | 2.86 |

Note: Each kilogram of milk powder contains 8 g of calcium, 6.6 g of phosphorus, 54 μg of selenium, 7.6 mg of manganese, 4 mg of copper, 18.1 mg of zinc, 0.179 mg of iodine, 20.5 mg of magnesium, 1.5 g of potassium, and 12.8 mg of iron.

**Table S2.** Milk feeding schedule for newborn piglets.

| Age Period (d) | Feeding Volume per Meal (mL) | Daily Feeding Frequency |
| --- | --- | --- |
| 0-2 |  |  |
| 2-4 | 10 | 12 |
| 5-7 | 20 | 12 |
| 8-10 | 30 | 10 |
| 11-13 | 40 | 10 |
| 14-16 | 60 | 8 |
| 17-19 | 70 | 8 |
| 20-21 | 70 | 8 |

Note: The milk replacer was prepared fresh immediately before each feeding by dissolving 206 g of solid milk replacer powder in 1 L of warm deionized water (40 °C). From d2 to d4, piglets were fed 10 mL per feeding at 2 h intervals, for a total of 6-8 feedings per day. Beginning on d5, the volume per feed was increased by 10 mL every 2-3 days until a total daily volume of 560 mL was reached.

**Table S3.** Primer sequences and accession numbers of genes used in qRT-PCR(pig).

| Gene（pig） | Primer sequences (5′-3′) | Accession numbes |
| --- | --- | --- |
| *GAPDH* | CGTCCCTGAGACACGATGGT | AF017079.1 |
|  | GCCTTGACTGTGCCGTGGAAT |  |
| *HAMP* | GAGCGAGTCCTAGAGCTAACA | NM_214117.1 |
|  | GGAAGTGGGTGTCTCTTCTT |  |
| *SOD* | AGATTCTGTGATCGCCCTCT | NM_001190422.1 |
|  | TCCAGCATTTCCCGTCTTTG |  |
| *CAT* | CGTTCTGTAAGGCTAGTCGG | XM_021081498.1 |
|  | AGGAATTCTCTCCCGGTCAA |  |
| *GPX* | GGGAGATCCTGAATTGCCTC | NM_214201.1 |
|  | TCGAAGTTCCATGCGATGTC |  |
| *NRF2* | TGACAGTGGCTGGTTGG | XM_003133500.6 |
|  | TCTCGACTTACTCCAAGGTC |  |
| *KEAP1* | CACGCTGCGATGGAGG | NM_001114671.1 |
|  | GTGTAGCTAAAGGTGCGGTT |  |
| *HMOX1* | GAACTTTCAGAAGGGCGAGG | NM_001004027.1 |
|  | GTAGACCGGGTTCTCCTTGT |  |
| *IL1Β* | GCTGATGGCCCCAAAGAGAT | NM_001302388.2 |
|  | TGCCACAATCACAGACACCA |  |
| *IFNG* | GCTTTGCGTGACTTTGTGTT | [NM_213948.1](https://www.ncbi.nlm.nih.gov/nuccore/NM_213948.1) |
|  | CTCCTTTGAATGGCCTGGTT |  |
| *TNF* | TCCAATGGCAGAGTGGGTATG | NM_214022.1 |
|  | AGCTGGTTGTCTTTCAGCTTCAC |  |
| *NOS2* | CCAGGCAATGGAGAGAAACT | NM_001143690.1 |
|  | CCGAACACAGCATACCTGAA |  |
| *CD86* | GTTCCTATCCACCAGATGAGT | NM_214222.1 |
|  | GAAGAGACACCCTGATTGATAC |  |
| *IL6* | AAATGTCGAGGCTGTGCAGA | NM_214399.1 |
|  | TCCACTCGTTCTGTGACTGC |  |
| *IL12* | TGCAGGGGTGGTGGTAATTC | NM_214013.1 |
|  | GGCATTAAGGAGCAACCAGC |  |
| *IL4* | CTCACCTCCCAACTGATCCC | NM_214123.1 |
|  | CTCCATGCACGAGTTCTTTCTC |  |
| *IL10* | ACCAGATGGGCGACTTGTTG | NM_214041.1 |
|  | TCTCTGCCTTCGGCATTACG |  |
| *MRC1* | GCCCAGACTGAAGACAGCAT | JN989538.1 |
|  | GGCATCTACCAGGCAGTTGT |  |
| *ARG1* | AGACCACAGTATGGCGATTG | XM_005659191.1 |
|  | AGTGTTGATGTCGGTGTGAG |  |
| *TLR4* | TCAGTTCTCACCTTCCTCCTG | GQ503242.1 |
|  | GTTCATTCCTCACCCAGTCTTC |  |
| *MYD88* | GATGGTAGCGGTTGTCTCTGAT | AB292176.1 |
|  | GATGCTGGGGAACTCTTTCTTC |  |
| *NFKBIΑ* | TGTTGGTGTCTTTGGGTGCT | NM_001005150.1 |
|  | GACATCAGCCCCACACTTCA |  |
| *RELA* | TACTGATGAGGACCTGGGGG | NM_001114281.1 |
|  | ATACACCCTGGTTCAGCAGC |  |

**Table S4.** Primer sequences and accession numbers of genes used in qRT-PCR(mouse).

| Gene（mouse） | Primer sequences (5′-3′) | Accession numbes |
| --- | --- | --- |
| *Gapdh* | GGCAAATTCAACGGCACAGT | NM_001289726.1 |
|  | AGATGGTGATGGGCTTCCC |  |
| *Il1b* | TTCAGGCAGGCAGTATCACTC | NM_008361.4 |
|  | GAAGGTCCACGGGAAAGACAC |  |
| *Tnf* | GACGTGGAACTGGCAGAAGAG | NM_013693.3 |
|  | GACGTGGAACTGGCAGAAGAG |  |
| *Il6* | TAGTCCTTCCTACCCCAATTTCC | NM_031168.2 |
|  | TTGGTCCTTAGCCACTCCTTC |  |
| *Il12* | ATCATCAAACCAGACCCGCC | NM_001303244.1 |
|  | GAGGAACGCACCTTTCTGGTT |  |
| *Il4* | GGTCTCAACCCCCAGCTAGT | NM_021283.2 |
|  | GCCGATGATCTCTCTCAAGTGAT |  |
| *Il10* | AGCCTTATCGGAAATGATCCAGT | NM_010548.2 |
|  | GGCCTTGTAGACACCTTGGT |  |
| *Il1rn* | GCTCATTGCTGGGTACTTACAA | XM_006497727.4 |
|  | CCAGACTTGGCACAAGACAGG |  |
| *Tlr4* | ATGGCATGGCTTACACCACC | NM_021297.3 |
|  | GAGGCCAATTTTGTCTCCACA |  |
| *Myd88* | TCATGTTCTCCATACCCTTGGT | NM_010851.3 |
|  | AAACTGCGAGTGGGGTCAG |  |
| *Nfkbiα* | AGACTCGTTCCTGCACTTGG | NM_010907.2 |
|  | AGGGGGAGTAGCCTTGGTAG |  |
| *Rela* | GGCTACACAGGACCAGGAAC | NM_009045.5 |
|  | GCCTGGTCCCGTGAAATACA |  |

**Table S5.** Details of antibodies.

| Antibodies | Cat No. | Source | Dilution |
| --- | --- | --- | --- |
| GAPDH | 60004-1 | Proteintech, Chicago, USA | 1:50000 |
| FTH | Bs-8679R | Bioss, Beijing, China | 1:1000 |
| GRP78 | A0241 | Abclonal | 1:1000 |
| p-PERK | AP1501 | Abclonal | 1:1000 |
| PERK | A8196 | Abclonal | 1:1000 |
| p-IRE1α | AP0878 | Abclonal | 1:1000 |
| IRE1α | F0229 | Selleck chemicals, Houston, USA | 1:500 |
| ATF6 | A0202 | Abclonal | 1:500 |
| ATF4 | A18687 | Abclonal | 1:1000 |
| CHOP | A0221 | Abclonal | 1:5000 |
| MyD88 | AF7524 | Beyotime | 1:1000 |
| p-IκBα | AF2002 | Affinity, Cincinnati, OH, USA | 1:1000 |
| IκBα | 10268-1-AP | Proteintech, Chicago, USA | 1:5000 |
| p-NF-κB P65 | AF2006 | Affinity, Cincinnati, OH, USA | 1:1000 |
| NF-κB P65 | 10745-1-AP | Proteintech, Chicago, USA | 1:2000 |


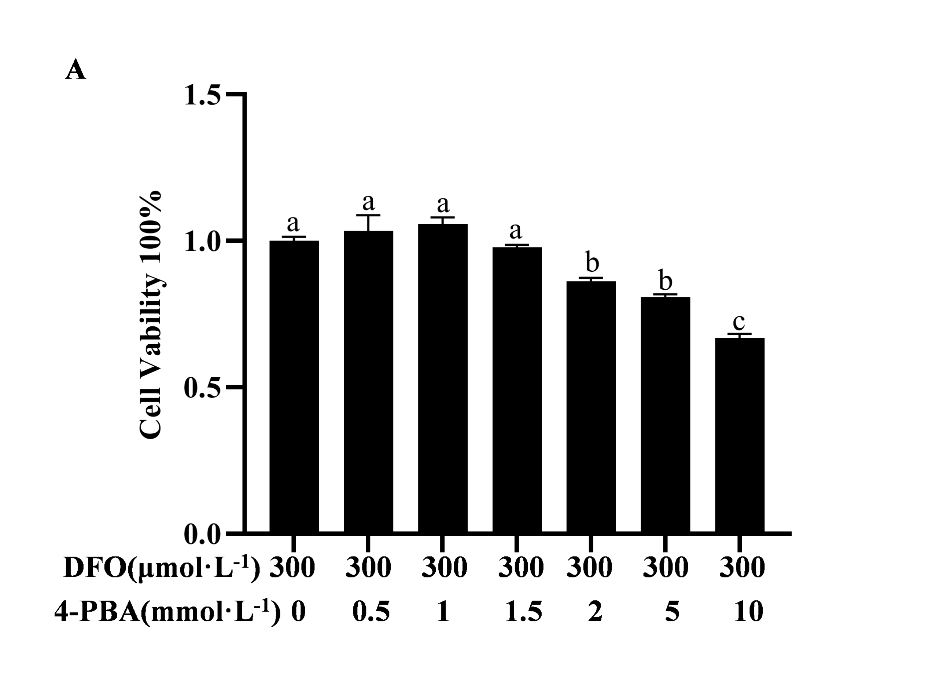


**Fig. S1A.** Cell viability was detected by CCK-8 assay in AML12 cells treated with 4-PBA and DFO.
