# Supplementary figures and images for: Iron deficiency aggravates hepatic inflammation in suckling piglets via endoplasmic reticulum stress-driven NF-κB pathway activation
Source: J Anim Sci Biotechnol. 2026 Feb 13;17:30. doi: 10.1186/s40104-026-01356-4 (PMC12903631; doi:10.1186/s40104-026-01356-4)

Fig 1B

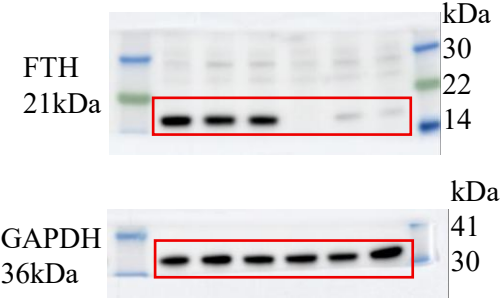

Fig 2C

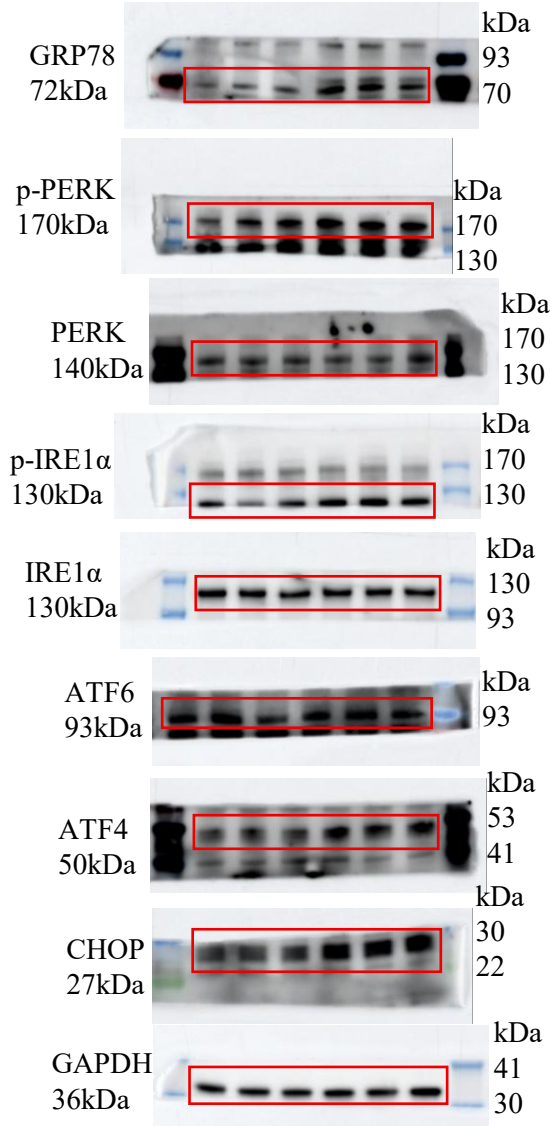

Fig 3I

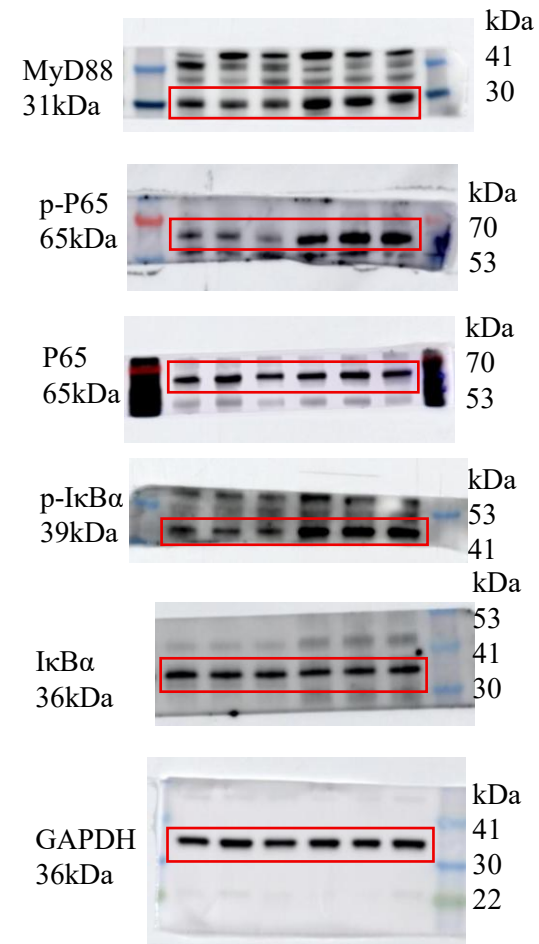

Fig 4D

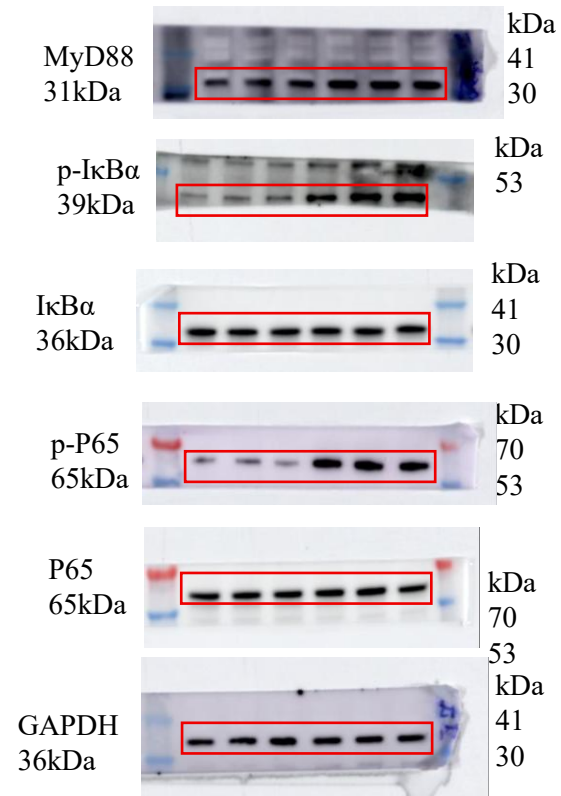

Fig 4H

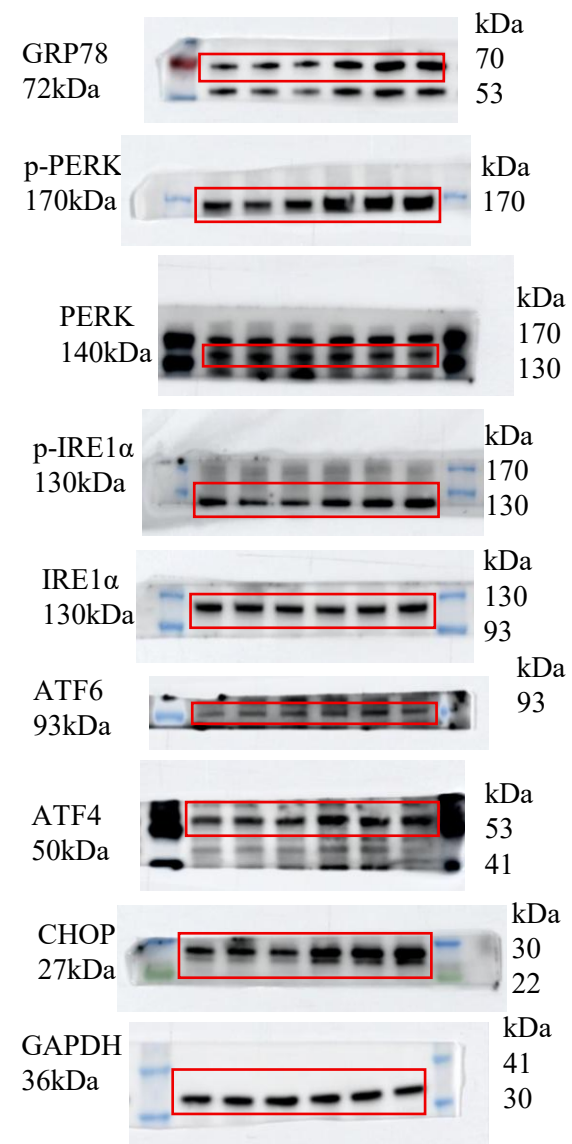

Fig 5A

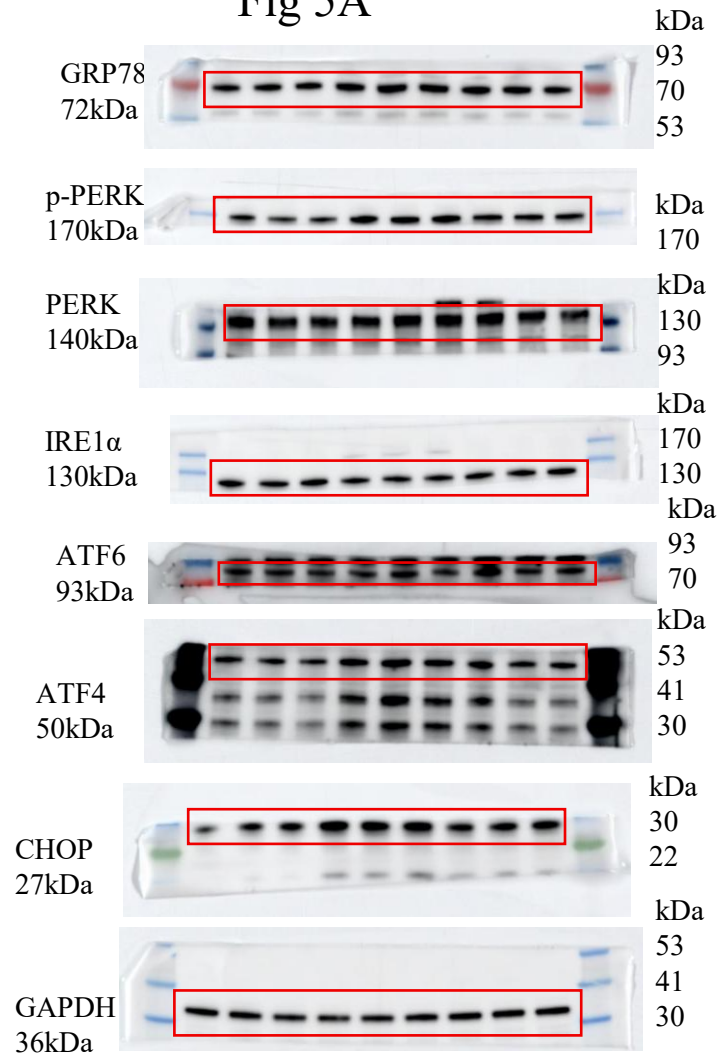

Fig 5K

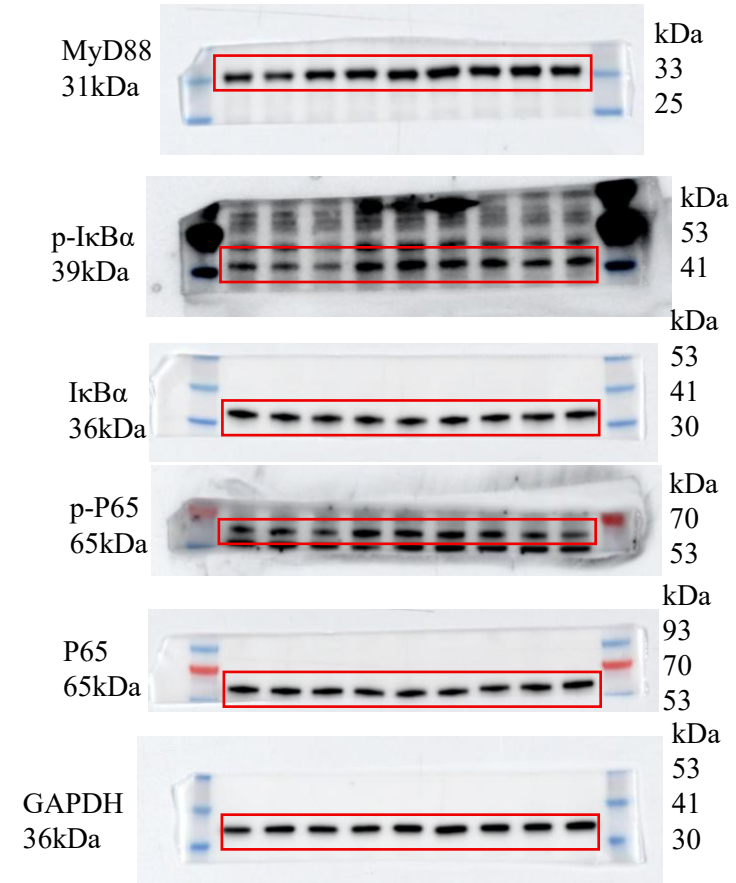

Supplement: Supplementary file 2 — Additional file 2. Original Western blot images. [file 40104_2026_1356_MOESM2_ESM.pdf]
